# Epigenetic modifiers upregulate MHC II and impede ovarian cancer tumor growth

**DOI:** 10.18632/oncotarget.17395

**Published:** 2017-04-24

**Authors:** Taylor B Turner, Selene Meza-Perez, Angelina Londoño, Ashwini Katre, Jacelyn E. Peabody, Haller J. Smith, Andres Forero, Lyse A Norian, J. Michael Straughn, Donald J. Buchsbaum, Troy D. Rand all, Rebecca C. Arend

**Affiliations:** ^1^ Department of Obstetrics and Gynecology, University of Alabama at Birmingham, Birmingham, AL, USA; ^2^ Division of Clinical Immunology and Rheumatology, University of Alabama at Birmingham, Birmingham, AL, USA; ^3^ Department of Radiation Oncology, University of Alabama at Birmingham, Birmingham, AL, USA; ^4^ NIH Medical Scientist Training Program, University of Alabama at Birmingham, Birmingham, AL, USA; ^5^ Department of Medicine, University of Alabama at Birmingham, Comprehensive Cancer Center, Birmingham, Alabama, USA; ^6^ Department of Nutrition Sciences, University of Alabama at Birmingham, Birmingham, AL, USA

**Keywords:** epigenetics, ovarian cancer, MHC II, HDACi, DNMTi

## Abstract

Expression of MHC class II pathway proteins in ovarian cancer correlates with prolonged survival. Murine and human ovarian cancer cells were treated with epigenetic modulators – histone deacetylase inhibitors and a DNA methyltransferase inhibitor. mRNA and protein expression of the MHC II pathway were evaluated by qPCR and flow cytometry. Treatment with entinostat and azacytidine of ID8 cells *in vitro* increased mRNA levels of *Cd74*, *Ciita*, and *H2-Aa*, *H2-Eb1*. MHC II and CD74 protein expression were increased after treatment with either agent. A dose dependent response in mRNA and protein expression was seen with entinostat. Combination treatment showed higher MHC II protein expression than with single agent treatment. In patient derived xenografts, *CIITA*, *CD74*, and MHC II mRNA transcripts were significantly increased after combination treatment. Expression of MHC II on ovarian tumors in MISIIR-Tag mice was increased with both agents relative to control. Combination treatment significantly reduced ID8 tumor growth in immune-competent mice. Epigenetic treatment increases expression of MHC II on ovarian cancer cells and impedes tumor growth. This approach warrants further study in ovarian cancer patients.

## INTRODUCTION

Immunotherapy has transformed cancer treatment in several malignancies over the last decade. Augmenting pre-existing immune responses or blocking immune inhibition has shown improvements in overall survival in melanoma [1], multiple myeloma [2], and renal-cell carcinoma [3]. Recently its use has been extended to less immunogenic tumors, such as non-small cell lung cancer [4]. Epithelial ovarian cancer (OVCA) is the most lethal of the gynecological malignancies with an overall 5 year survival around 45% despite good initial response rates to chemotherapy [5]. While OVCA is not highly immunogenic, increased levels of immune effector cells within the tumor correlates with better anti-tumor responses and improved survival [6–8].

Epigenetic modulation, such as histone deacety-lation and DNA methylation, produces non-sequence DNA changes that affect transcription and translation. Epigenetic agents are known to impact anti-tumor immunity [9]. Histone deacetylase inhibitors (HDACi) change the acetylation patterns of lysines in histones and alter the chromatin structure by inhibiting the histone deacetylase enzyme [10]. There are 18 HDACi currently identified, and they are split into four categories based on sequence homology to yeast homologs. Classes I, II, and IV are more commonly targeted for inhibition, and class III is distinguished by NAD^+^ dependence [11]. Four HDACi are currently FDA-approved for lymphoma and/or multiple myeloma. Panobinostat is a non-selective HDACi that is FDA-approved for the treatment of multiple myeloma. Entinostat, which primarily targets class 1 HDACs, has been granted breakthrough therapy designation by the FDA for patients with advanced breast cancer. HDACi have been shown to activate antitumor immunity, including the major histocompatibility complex 2 (MHC II) pathway [12–16].

Two DNA methyltranferase inhibitors (DNMTi) are FDA-approved for treatment of myelodysplastic syndromes: azacytidine (5-azacytidine or 5AZA) and decitabine. Both are cytidine analogs that block the function of DNMT and facilitate its degradation. Inhibiting DNMT effects the activation or inactivation of gene expression via promoter methylation, apoptosis, and other changes in cell signaling [17]. Azacytidine has been shown to increase the expression of genes in several immunoregulatory pathways, including the MHCII pathway in OVCA [18]. MHC II is expressed on antigen presenting cells (APCs) such as dendritic cells, B cells, and macrophages, and activates naïve CD4^+^ T cells. The MHC II antigen-presenting pathway can be expressed by some tumor cells, and higher MHC II expression correlates positively with tumor infiltrating lymphocytes and overall survival in OVCA [19]. Endogenous histone acetylation and DNA methylation both cause a decrease in the expression of a key class II major histocompatibility complex transactivator (*CIITA*) [20]. We investigated the effect of combined inhibition of DNA methylation and histone acetylation on induction of MHC II pathway expression in OVCA tumor cells, with the goal of determining if this impaired ovarian tumor growth in immune-competent mice.

## RESULTS

### Dose selection of entinostat, panobinostat, and azacytidine in cell lines

**Figure 1:**
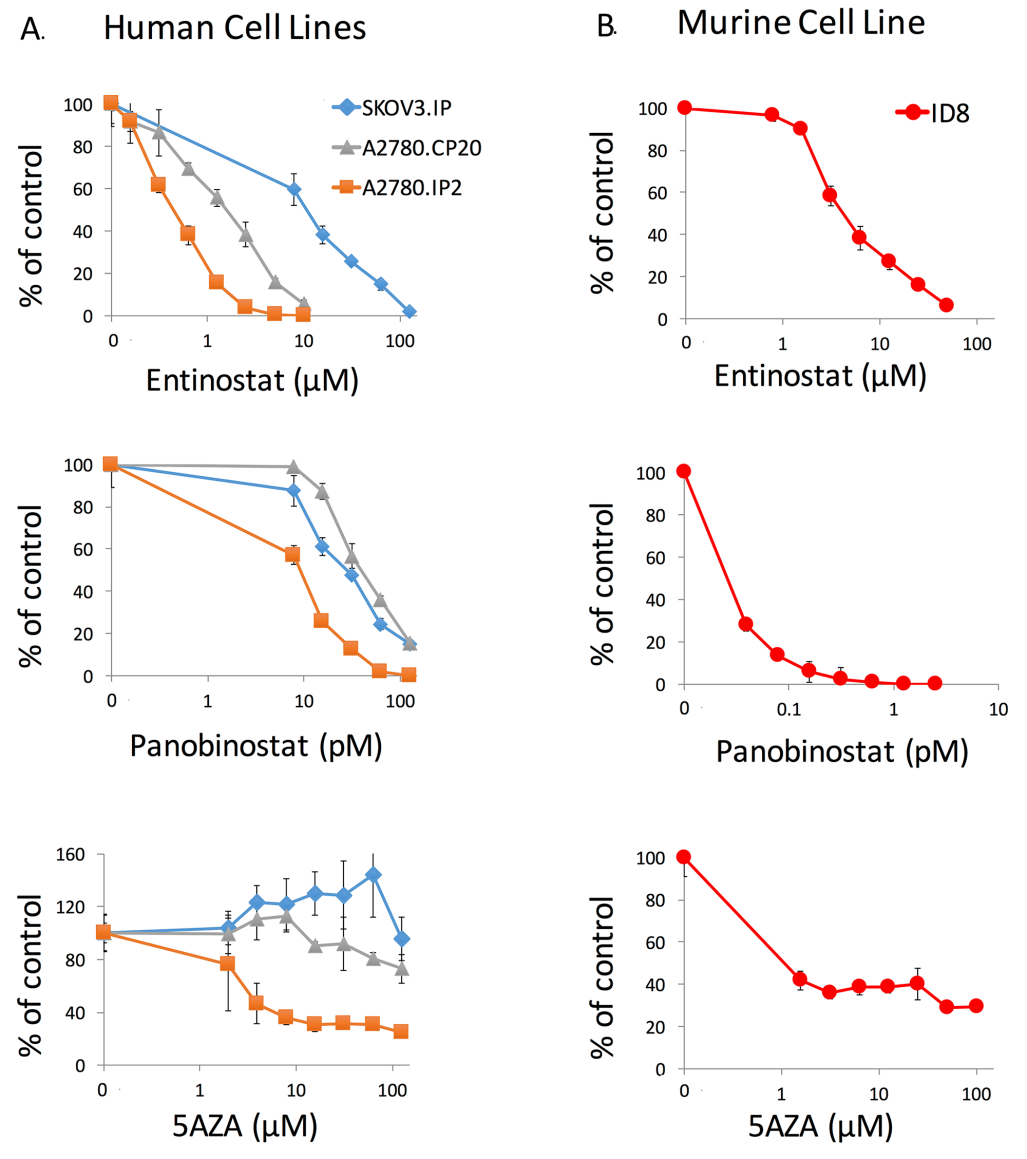
ATPlite assay of entinostat, panobinostat, and azacytidine against human and murine ovarian cancer cell lines **(A)** A2780.IP2, A2780.CP20, and SKOV3. IP were treated for 72 hours to identify a dose range using the ATPlite assay. **(B)** ID8 cells were treated for 72 hours with the same agents. Error bars represent standard error of the mean (SEM) for the eight replicates.

To evaluate effects of HDACi and DNMTi on MHC II pathway upregulation and growth of OVCA tumors, we focused our efforts on the agents panobinostat, entinostat and azacytidine to facilitate future translatability. To select appropriate doses of epigenetic modifiers for use in evaluating effects on the MHC II pathway, ATPlite assays were performed on both the ID8 murine cell line and three human OVCA cell lines: SKOV3.IP, A2780.CP20, A2780.IP2. Our goal was to identify the doses that impact expression of MHC II pathway genes while exhibiting limited cellular and animal toxicities. ATPlite assays demonstrated a dose range decrease in ATP production with entinostat, panobinostat, and azacytidine (Figure 1). Entinostat had consistent decrease in ATP with a range of IC_50_ values from 0.57 to 5.8μM in human cell lines, and 4.7μM in ID8 cells. The IC_50_ doses for panobinostat were 3.8, 19.8, and 62.4 pM for A2780.IP2, A2780.CP20, and SKOV3.IP respectively. For ID8 cells the IC_50_ was 0.03 pM. After azacytidine treatment, no decrease in ATP production was seen in the SKOV3.IP cell line at 62 μM, and 125 μM caused only a 5% decrease in ATP. However, azacytidine caused a reduction of ATP in A2780.IP2 cells, with an IC_50_ of 6.4 μM. A dose of 1.5 μM azacytidine caused 60% reduction of ATP in the ID8 cell line, with no increased effect at higher doses (Figure 1).

### Quantitative PCR demonstrates increase in MHC-II pathway transcript expression

**Figure 2:**
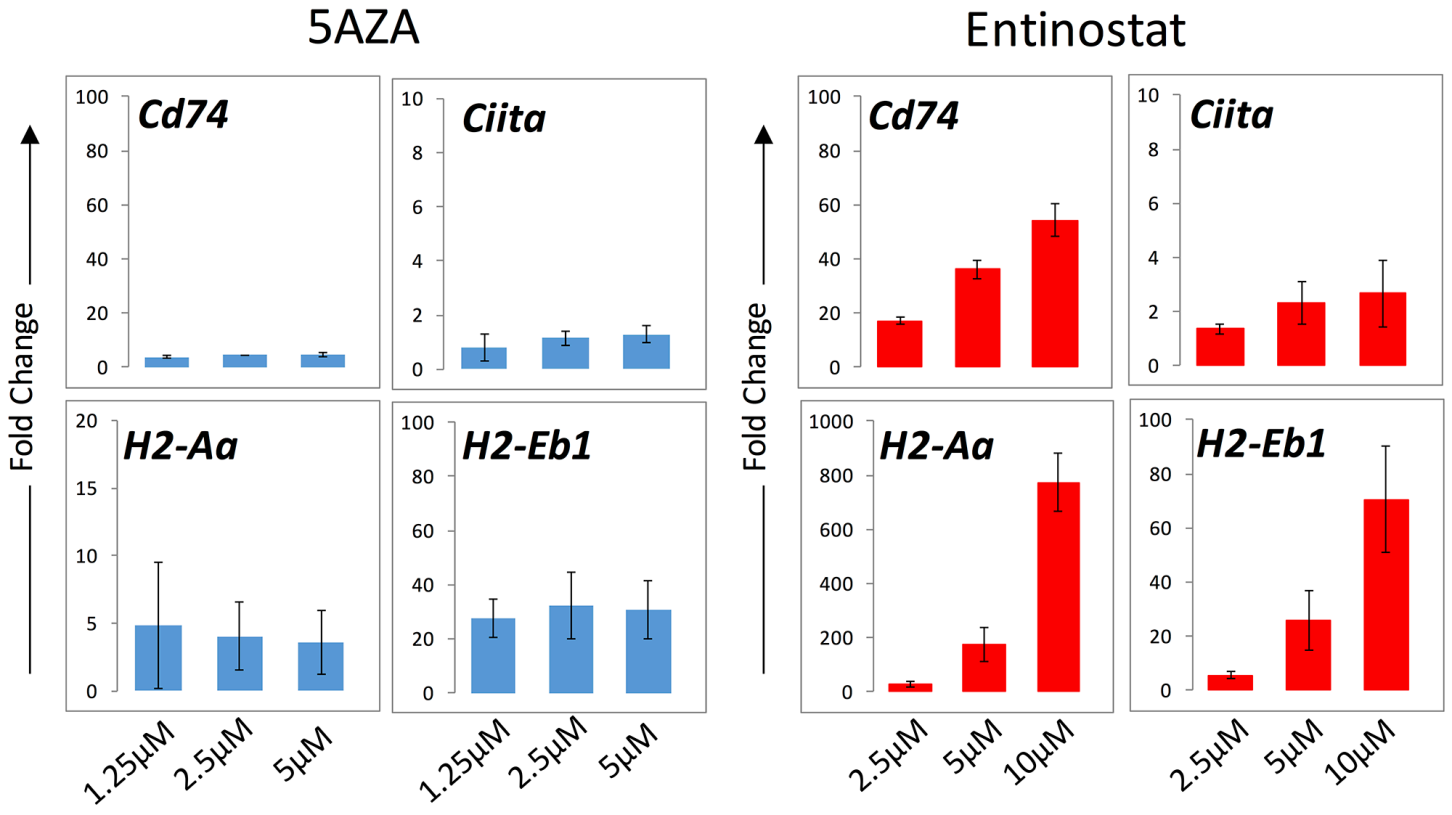
mRNA transcript levels of MHC II pathway genes in ID8 cells evaluated by qPCR Cells were exposed to entinostat and azacytidine for 24 hours at three concentrations. Bars represent median ± standard deviation of fold change across three replicates. The y axis represents fold change relative to untreated cells.

To test our hypothesis that epigenetic therapy increases expression of genes associated with the MHC II pathway, quantitative PCR was used to measure mRNA transcript levels of four components of the MHC II pathway in the ID8 cell line after treatment with a DNMTi and a HDACi. These included two components of the MHC II molecule, *H2-Aa* and *H2-Eb1*, the transactivator *Ciita*, and *Cd74*. *Cd74* is an intracellular member of the MHC II pathway that functions to stabilize MHC II after translation and directs it to the endoplasmic reticulum [21]. Entinostat increased *Cd74*, *H2-Aa*, *H2-Eb1*, and *Ciita* transcription in a dose-dependent fashion after 24 hours of exposure (Figure 2). After 24 hours of azacytidine treatment, there was a smaller increase in all four transcripts and no dose-dependent response was seen (Figure 2). After 72 hours of entinostat exposure, all four transcripts were increased relative to untreated control, and *Cd74* increased in a dose-dependent fashion (data not shown).

### HDACi and DNMTi increase expression of MHC II pathway proteins

**Figure 3:**
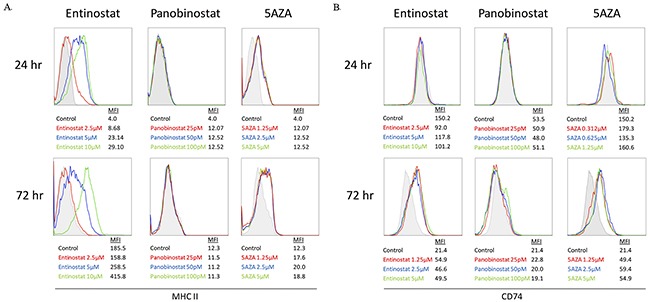
MHC II pathway upregulation on ID8 cells treated with entinostat, panobinostat, or azacytidine evaluated via flow cytometry **(A)** Histograms of MHC II expression after 24 and 72 hours of exposure are shown with mean fluorescence intensity (MFI) for each concentration. **(B)** Histograms of CD74 expression after 24 and 72 hours of exposure are shown with MFI for each concentration.

ID8 cells were exposed to entinostat, panobinostat, and azacytidine for 24 or 72 hours and MHC II pathway protein expression was evaluated by flow cytometry. Entinostat showed a temporal and dose-dependent increase in MHC II expression in ID8 cells, whereas no dose-dependent response was seen with azacytidine (Figure 3A). After 72 hours of panobinostat exposure, expression levels of MHC II were slightly lower than untreated controls in the ID8 cells with no dose-dependent response at either time point (Figure 3A).

Figure 3B shows an increase in CD74 expression, but only after 72 hours of treatment with azacytidine or entinostat in the ID8 cell line. Panobinostat produced no change in CD74 after 24 or 72 hours. Although panobinostat showed a dose-dependent decrease in ATP, it did not increase MHC II pathway expression at the selected doses; therefore, it was not used in our *in vivo* experiments. Entinostat showed both a dose-dependent reduction in ATP and an increase in MHC II and CD74 expression over time; therefore, it was used for HDAC inhibition in all additional experiments.

### Combination HDACi and DNMTi treatment has an additive effect on MHC II expression in ID8 cells

**Figure 4:**
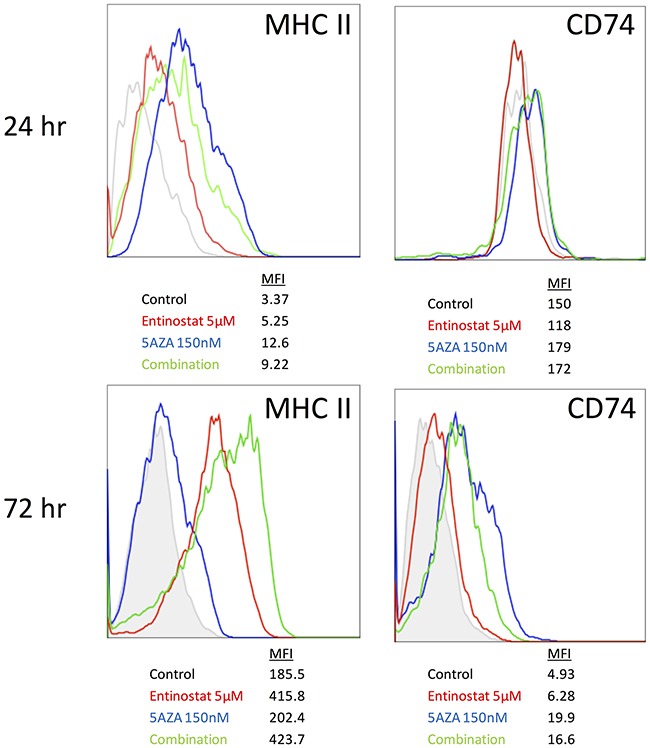
MHC II and CD74 protein expression on ID8 cells after combination treatment with entinostat and azacytidine MHCII and CD74 expression were measured by flow cytometry 24 and 72 hours after treatment with 5 μM entinostat in combination with 150 nM azacytidine.

In order to investigate the combined effect of entinostat and azacytidine on ID8 cells, we measured MHC II and CD74 protein expression by flow cytometry. After 24 hours of treatment azacytidine alone and combination treatment showed similar increase in MHC II expression. However, after 72 hours of treatment, MHC II expression was greater with the combination of azacytidine plus entinostat than with either agent alone (Figure 4). In contrast, CD74 expression did not increase with the combination of entinostat plus azacytidine, relative to azacytidine alone, at either 24 or 72 hours.

### *In vivo* mRNA response in a patient derived xenograft (PDX) model

**Figure 5:**
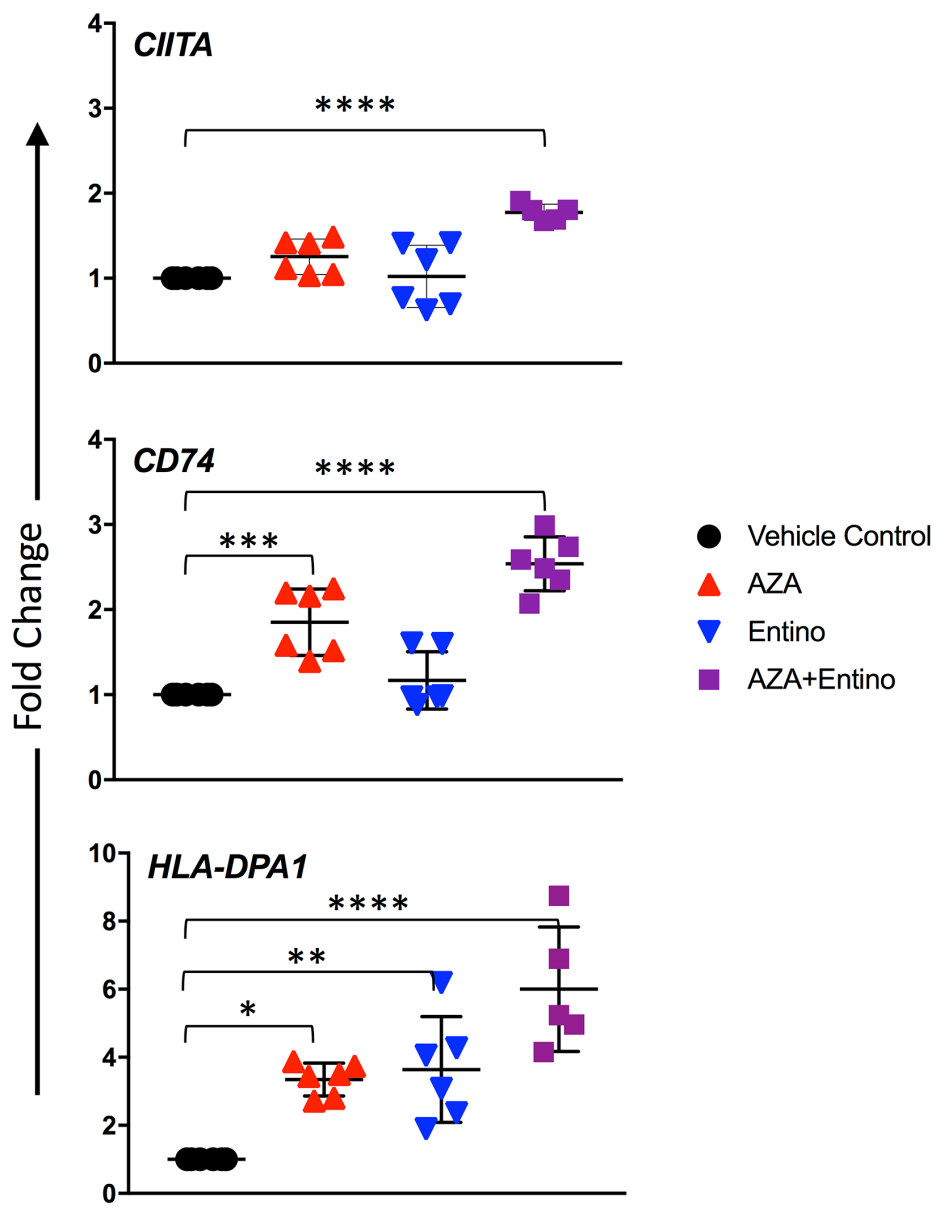
Increased mRNA expression of the MHC pathway in a PDX model mRNA transcript levels after 2 weeks of treatment in ovarian PDX tumors implanted subcutaneously. Doses were daily azacytidine 2mg/kg, entinostat 5mg/kg, and both agents in combination. Data represent three technical replicates per animal in each treatment group with SD. Values compared to control were considered significant when P < 0.05. * p ≤ 0.05; ** p ≤ 0.01, *** p ≤ 0.001; **** p ≤ 0.0001.

To determine if the combination of entinostat plus azacytidine led to increased MHC II gene expression *in vivo*, we began by evaluating an OVCA PDX model in SCID mice. *CIITA* was significantly increased (1.8-fold, p < 0.0001) relative to the vehicle control group in the combination treatment group, and the mean difference in fold change was significantly higher than in mice treated with azacytidine (p = 0.005) or entinostat alone (p = 0.0001) (Figure 5). In response to the combination treatment, *CD74* expression was increased 2.5-fold (p < 0.0001) relative to vehicle controls, which was significant as compared to azacytidine or entinostat alone (p = 0.004) and (p = 0.0001), respectively. Treatment with only azacytidine resulted in a significant 1.9-fold increase (p = 0.0005) of *CD74* relative to the vehicle control group, whereas *CD74* expression was not statistically different in the entinostat group.

MHC class II antigen DP Alpha 1 (*HLA-DPA1*) is a MHC class II paralogue. Single nucleotide polymorphisms within *HLA-DP* have been significantly associated with OVCA incidence and increased tumor aggressiveness [22]; therefore, we selected this locus to evaluate. Treatment with azacytidine or entinostat resulted in significant increases in *HLA-DPA1* expression relative to the vehicle group (p = 0.0135 and 0.0053, respectively). PDX mice treated with both azacytidine and entinostat had an even higher expression of *HLA-DPA1* relative to vehicle control, with a 6-fold increase.

### *In vivo* protein response in the MISIIR-Tag OVCA model

**Figure 6:**
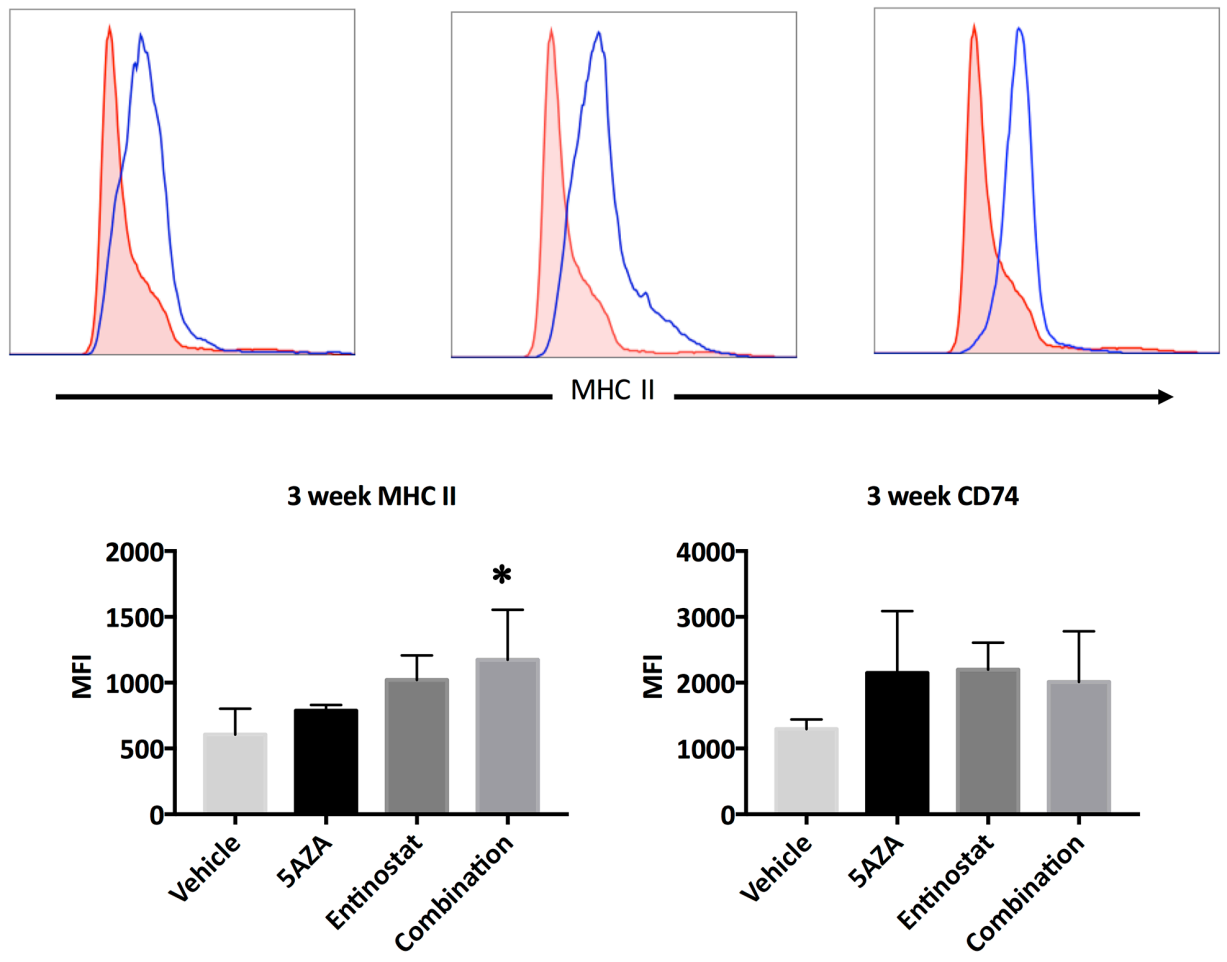
Expression of MHC II and CD74 proteins in ovarian tumors of MISIIR-Tag mice after 3 weeks of treatment All mice initiated treatment at 10 weeks of life. Doses were daily azacytidine 2 mg/kg, entinostat 5 mg/kg, and both agents in combination. Histograms and bar graphs depict MHC II and CD74 expression on gated CD45-negative single cells within dissociated tumors. Dunnett's test comparing combination treatment to vehicle control show a significant difference (p = 0.0357), marked with *.

The immunoregulatory effect of a DNMTi in a syngeneic murine OVCA model was recently shown by Wang *et al*., but the effects of epigenetic therapy in a spontaneously developing OVCA mouse model have never been described [23]. The development of ovarian carcinomas and spread to peritoneal organs has been described by Connolly and colleagues in the MISIIR-Tag mice developed in their laboratory, but little has been described about the life span of these mice [24]. In our experience, these mice develop ovarian tumors by 10 weeks of life, many develop ascites, and few survive past 25 weeks. We sought to evaluate the effect of HDACi and DNMTi treatment in this *in vivo* model. Therefore, MISIIR-Tag mice were treated with azacytidine, entinostat, the combination of both agents, or vehicle control starting at the time tumors first develop (10 weeks of age). Tumor cell expression of MHC II and CD74 were evaluated by flow cytometry after 3 weeks of treatment. Hematopoetic cells within tumors were excluded by CD45 staining. The combination drug treatment resulted in a statistically significant increase in MHC II expression on the surface of CD45- cells within ovarian tumors, as compared to vehicle control (p = 0.0357) (Figure 6). This increase was not observed when the two agents were used independently, supporting our hypothesis that the combination of both epigenetic modifiers enhances the upregulation of MHC II expression on OVCA cells. Treatment with azacytidine, entinostat, or the combination resulted in a trend toward increased CD74 expression that was not statistically significant (Figure 6).

### Effects of combination therapy on ovarian tumor growth

**Figure 7:**
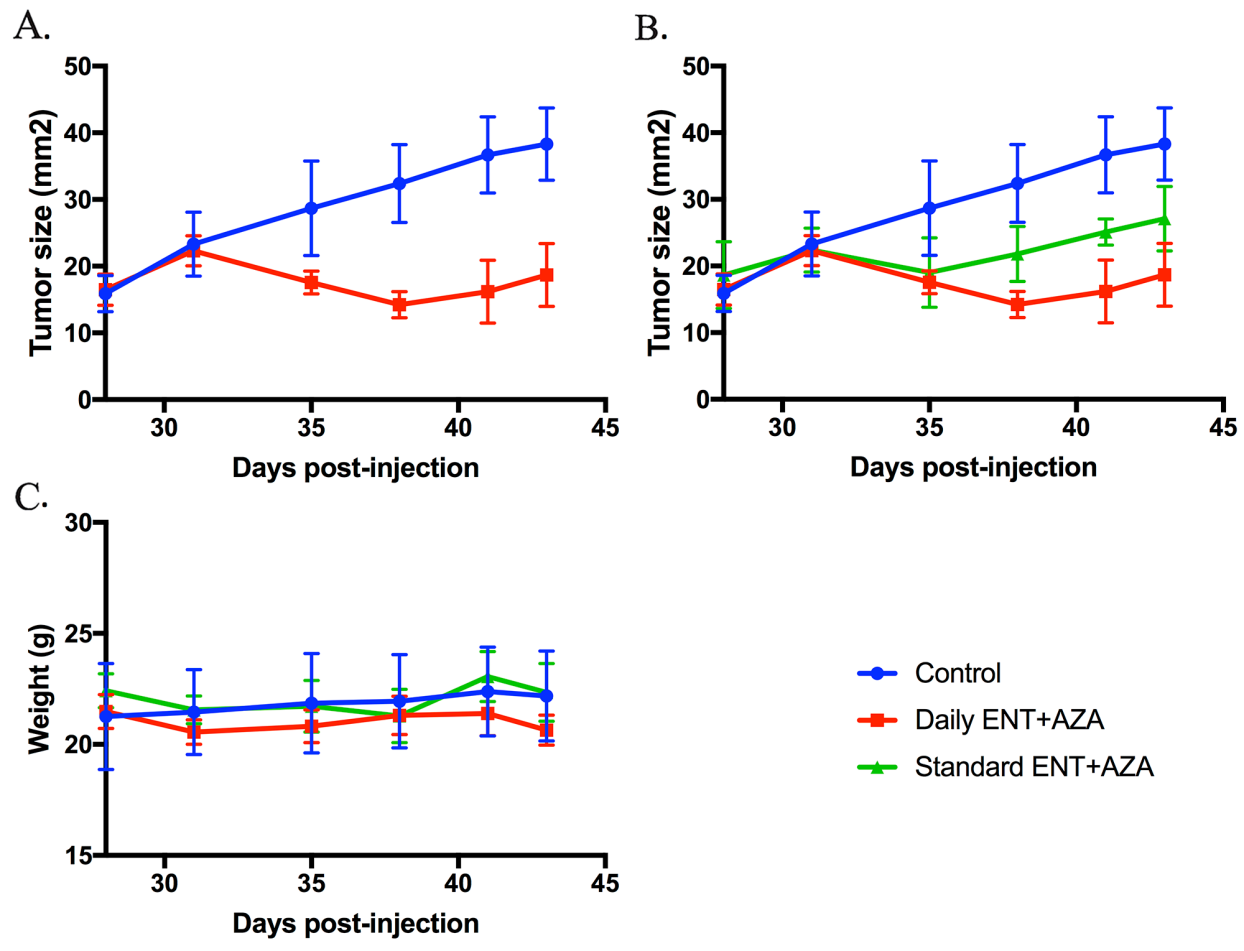
Tumor size and mouse weights after 15 days of treatment with combination HDACi and DNMTi therapy ID8 C57BL/6 mice were treated with entinostat and azacytidine. Doses were entinostat 20 mg/kg daily and every other day (standard) and azacytidine 0.8mg/kg daily. Tumor size in both the daily group **(A)** and the standard treatment group **(B)** were significantly reduced compared to untreated controls (both p < 0.05). Treatments were well-tolerated with no significant weight loss occurring in any group **(C)**.

Finally, we sought to determine if the combination of entinostat and azacytidine was able to impair ovarian tumor growth in immune-competent mice. Although the spontaneous MIISR mouse model has many advantages, the nature of this model makes it difficult to evaluate treatment effects on tumor growth. For this reason, we implanted ID8 OVCA cell in syngeneic C57BL/6 mice. Mice were treated with one of two combination regimens: either daily treatments of both entinostat and azacytidine or a regimen where entinostat was given every other day in combination with daily azacytidine. After 15 days of treatment, tumor sizes in the daily treatment group were significantly reduced as compared to untreated control mice (Figure 7). The mean tumor area for the control group was 38 mm^2^, whereas the daily combination treatment group had a mean tumor size of 19 mm^2^ (p = 0.0001). Every other day treatment resulted in a mean of 27 mm^2^, which was also significantly smaller than untreated control tumors (p = 0.011). The difference between the two treatment regimens at 15 days approached statistical significance (p = 0.051). Importantly, mouse weights during treatment showed no significant differences between controls and treatment groups, illustrating that the combination treatment was well-tolerated.

## DISCUSSION

In this study, we evaluated the effects of two types of epigenetic modifiers on human OVCA cell lines, murine cell lines, PDX, and in two immune-competent OVCA murine models. While the PDX model cannot be used to show an immune response, we wanted to replicate what we observed in the OVCA cell lines *in vitro* in an *in vivo* model with a patient dervied ovarian tumor. There was an additive increase of the MHC II pathway protein and gene expression with the combination of entinostat and azacytidine treatment, specifically MHC II complex genes *CIITA* and *CD74*. This study demonstrates that the combination of entinostat plus azacytidine upregulates MHC II mRNA and protein expression in OVCA cells *in vitro* and tumors *in vivo*, and reduce primary tumor growth. Although epigenetic agents have been tested in OVCA human subjects without significant success [25, 26], previous trials tested a single agent. Here, we demonstrate a reduction in tumor growth without significant toxicity using two agents.

This study illustrates that epigenetic manipulation can upregulate of MHC II pathway gene and protein expression in both human and murine OVCA cells. Treatment with azacytidine and entinostat increased the mRNA and protein expression of MHC II as well as other components of the MHC II processing pathway. Panobinostat did not show the same effect, which may be related to its function as a pan-deacetylase inhibitor compared to entinostat that more specificically inhibits HDAC1 and 3. While HDACi and DNMTi likely alter the expression patterns of additional genes, the presence of MHC II on the cell surface of tumor cells may result in an increase in neoantigen presentation to the immune system. Epigenetic treatment has been shown to elicit an interferon response in cancer cells, including increased antigen presentation pathways [27, 28].

Treatment with the combination of azacytidine and entinostat has been evaluated in other pre-clinical cancer models. Kim *et al*. reported that treatment of colorectal and mammary syngeneic animal models with DNMTi and HDACi caused decreased tumor growth rate. When these tumors were treated with checkpoint inhibitors against PD-1 and CTLA-4, a period of tumor shrinkage followed by tumor growth was observed [29]. When all 4 therapies were given in combination, even large tumors were eradicated and no secondary growth was observed [29]. In that study, the mechanism behind the effect of HDACi and DNMTi was not clearly identified. We present data demonstrating that the combination of HDACi and DNMTi increases the expression of the MHC II antigen-presentation pathway genes. This could explain the synergy of checkpoint inhibitors and epigenetics as seen in the study by Kim *et al*. This theory is supported clinically in melanoma where MHC II expression predicted response to anti-PD-1/PD-L1 therapy [30]. This will need to be tested experimentally in future studies in syngeneic OVCA models.

This study has several limitations. There is significant debate regarding the genomics of OVCA cell lines, and genetic analysis does not always correlate with *in vivo* tumor histology [31, 32]. As such, cell line data alone is of limited utility, but in combination with *in vivo* models including PDX models, cell line results can add to overall understanding. We demonstrated increased MHC II RNA and protein expression but did not confirm the functional antigen presentation of MHC II. Finally, while there was an encouraging reduction in tumor growth with the combination treatment, this could be a direct cytotoxic effect as we did not attempt to correlate with the immune response. Future studies will determine if these responses are linked to enhanced T-cell mediated responses.

Others have reported on the use of HDACi and DNMTi in tumor models, and their ability to impact anti-tumor immune responses, but we demonstrate a specific mechanism that has not been previously described in OVCA [13–15, 18]. The increase in expression of MHC II pathway proteins in OVCA tumor cells provides a possible rationale for how epigenetic therapy could augment the immune system's anti-tumor response, and provides support for the future investigation of these agents in OVCA patients.

## MATERIALS AND METHODS

### Cell lines and culture conditions

ID8, a murine OVCA cell line, was obtained from Dr. Yancey Gillespie (University of Alabama at Birmingham, AL). The OVCA cell lines A2780.IP2 and A2780.CP20 (platinum resistant) were acquired courtesy of Dr. Charles Landen (University of Virginia, Charlottesville, VA). SKOV3.IP was acquired from the American Type Culture Collection (Manasses, VA). The cell lines were maintained in RPMI-1640 medium supplemented with 10% fetal bovine serum (Atlanta Biologicals, Flowery Branch, GA). All cell lines were routinely screened for *Mycoplasma* species (GenProbe detection kit; Fisher, Itasca, IL) with experiments performed at 70–80% confluent cultures. Purity of cell lines was confirmed with STR genomic analysis, and only cells less than 20 passages from stocks were used in experiments.

### ATPlite luminescence-based assay

Murine OVCA cell line ID8 and human OVCA cell lines A2780.IP2, A2780.CP20 (platinum resistant), and SKOV3.IP were plated in 96-well tissue culture plates (Corning Costar, NY) at 2000 cells/well in 45μl RPMI + 10% FBS. The plates were incubated at 37 °C in a humidified 5% CO_2_ atmosphere and treated on day 2 with a single dose of entinostat (LC Laboratories, Woburn, MA), panobinostat (LC Laboratories), azacytidine (TOCRIS, Bristol, UK) or entinostat combined with azacytidine. Testing was done in duplicate assays with 8 replicates each. Cells were collected after either 24 or 72 hours of drug exposure and cytotoxicity was evaluated using the ATPlite luminescence-based assay (PerkinElmer, Waltham, MA) as previously described [33].

### Quantitative polymerase chain reaction (qPCR) analysis

Murine OVCA ID8 cells were plated in RPMI + 10% FBS in 6-well cell culture plates (Corning Costar). The following day, cells were treated with a single application of entinostat, azacytidine and/or panobinostat alone or in combination. All agents were dissolved in DMSO and a DMSO-only well was used as control. Concentrations were tailored to minimize DMSO volume; in all treatments the DMSO concentration did not exceed 0.1%. Either 24 or 72 hours after treatment, cells were lifted with Accutase (Innovative Cell Technologies, San Diego, CA). Cells were split and processed for RNA extraction using RNeasy Plus Kit (Qiagen, Valencia, CA) according to the manufacturer's protocol. RNA was converted to cDNA using Superscript VILO Master Mix (Thermo Fisher, Waltham, MA) and qPCR was performed using Power SYBR Green PCR Master Mix (Life Technologies, Carlsbad, CA) on an Applied Biosystems 7900HT Fast Real-Time PCR System following the manufacturer's protocol. For patient derived xenograft (PDX) experiments, the same procedure was performed but on a CFX connect Real-Time System (Bio-Rad, Hercules, CA). All samples were amplified in triplicate, normalized to GAPDH, and fold change relative to untreated control was calculated as 2^(−ΔΔCT) [34]. Murine primers were specific for *H2-Aa*, *H2-Eb1*, *Gapdh*, *Ciita*, and *Cd74* (Sigma, St. Louis, MO). PDX tumor primers were specific to *GAPDH*, *CIITA*, *CD74* (Sigma), and *HLA-DPA1* (Integrated DNA Technologies, Coralville, IA).

### *In vivo* mouse models

All animal studies were approved by the University of Alabama at Birmingham Institutional Animal Care and Use Committee before they were started and were performed in compliance with national regulatory agency guidelines. Dr. Denise Connolly (Fox Chase Cancer Center, Philadelphia, PA) provided MISIIR-Tag mice that express SV40 T antigen oncogene under the Mullerian Inhibitory Substance type II Receptor promoter. These mice spontaneously develop ovarian tumors that invade the peritoneal cavity including the omentum [24]. The MISIIR-Tag mice were used to analyze entinostat and AZA treatment on MHCII pathway protein expression in a syngeneic spontaneous OVCA mouse model. For PDX experiments, under IRB approval, high grade papillary serous ovarian tumors were collected and implanted as previously described [35] and used for MHCII pathway mRNA expression analysis after treatment with entinostat and AZA in a model independent of the immune response. After processing there was minimal tissue so no protein expression analysis was performed. 7-week-old C57BL/6 mice were obtained from Charles River Laboratories (Wilmington, MA) and used for subcutaneous ID8 tumor challenge experiments.

### Flow cytometry

Cells from murine and human cell lines were plated and treated as above, and were collected and washed in PBS with 1% BSA and 0.1% NaN_3_ followed by staining with fluorochrome-conjugated antibodies. Murine lines were stained with conjugated anti-CD74 (In-1) and anti- I-A/I-E (M5/114) (BD Biosciences, San Jose, CA). Human cell lines were stained with conjugated anti-CD74 (LN2) and anti-HLA-DR, DP (Tu36) (BioLegend, San Diego, CA). Fixation and permeabilization were performed with BD Cytofix/Cytoperm (BD Biosciences). Murine MISIIR-Tag tissues were stained with conjugated anti-CD45.2 (104) (eBioscience, San Diego, CA) anti-IA/IE (IgG2b, κ) (BioLegend) and anti-CD74 (WF) (BD Biosciences). Fixation and permeabilization were performed with the Foxp3 / Transcription Factor Staining Buffer Set (eBioscience). MHC II and CD74 expression were evaluated on gated CD45-negative single cells within dissociated tumors. All samples were evaluated using a BD FACS CANTO II (BD Biosciences). FlowJo (FlowJo, Ashland, OR) v10.0.8r1 was used to analyze flow cytometry data.

### *In vivo* PDX and spontaneous ovarian cancer models

For the MISIIR model, mice were treated 5 days per week, starting at 10 weeks for 3 weeks. Each treatment group had three (MISIIR-Tag) mice. The PDX experiment consisted of groups of two mice treated for 2 weeks in the same fashion; the treatment period was shortened based on pilot experiments suggesting an earlier response in RNA expression changes. All treatment agents were dissolved in DMSO and diluted in a mixture of PBS and Kolliphor EL to aid in solubility. The final volume ratio of DMSO:Kolliphor EL:PBS was 1:2:7. Mice were treated with entinostat (5mg/kg), azacytidine (2mg/kg), a combination of both agents at the same concentrations, or vehicle-only control. Drug concentrations were made to create injection volumes of approximately 200μL. After treatment, MISIIR mice were euthanized and ovaries and tumor were collected. Ovary and tumor were cut into small pieces and shaken at 300 RPM at 37°C for 30 min in RPMI with 5% FBS, DNAse, and collagenase, then filtered in 70μm mesh and washed to isolate single cells for flow cytometry. For qPCR on PDX tumors the tumor tissue was snap-frozen in liquid nitrogen and stored at -80 °C before quantitative PCR analysis. Total RNA was isolated using an AllPrep DNA/RNA Mini Kit (Qiagen).

### Syngeneic ID8 mouse model

C57BL/6 mice were injected subcutaneously in the flank with 7×10^6^ ID8 cells, and treatments were started 28 days later with 5 mice per treatment group. Tumors were measured bi-weekly in two dimensions. Combination treatment with entinostat 20mg/kg and azacytidine 0.8mg/kg was used in two regimens. The daily regimen consisted of intraperitoneal injections of entinostat every day with every other day azacytidine, and the other regimen consisted of intraperitoneal injections of both drugs every other day. Mice were weighed twice weekly and examined daily for any evidence of adverse side effects.

### Statistical analysis

For ATPlite assays, the IC_50_ (half maximum inhibitory concentration) was defined as the log_10_ of the concentration producing 50% reduction in ATP levels (in counts per second) compared with the untreated cells. ATP production was measured using a ratio of ATP levels for treated cell lines to untreated controls (percent control). All data were imported into JMP Pro 12 for analysis and presented as mean +/− standard deviation (SD) or standard error (SE). Statistical significance for qPCR and flow cytometry was determined by ANOVA and *p* < 0.05 was considered significant. Mean fluorescence intensity (MFI) was calculated using raw data from flow cytometry. MFI was calculated for each sample and all groups were compared using ANOVA. Individual treatments were compared to controls using Dunnett's test. Tumor size was compared across treatment groups with ANOVA using the 28 day measurements.

## Abbreviations

OVCA: ovarian cancer
MHC II: major histocompatibility complex 2
HDACi: histone deacetylase inhibitors
DNMTi: DNA methyltransferase inhibitor
APC: antigen presenting cell
PDX: patient derived xenograft
